# Next-generation plant science: putting big data to work

**DOI:** 10.1186/gb4149

**Published:** 2014-01-15

**Authors:** Elizabeth K Brauer, Dharmendra K Singh, Sorina C Popescu

**Affiliations:** 1 The Boyce Thompson Institute for Plant Research, Ithaca, NY 14853, USA; 2 Department of Plant Pathology and Plant-Microbe Biology, Cornell University, Ithaca, NY 14853, USA

## Abstract

A Report on the Plant Genomes & Biotechnology: From Genes to Networks meeting, held at the Cold Spring Harbor Laboratories, USA, December 4–7, 2013.

## 

The introduction of next-generation sequencing has benefitted plant science due to a rapidly expanding number of fully sequenced and annotated plant genomes. The availability of genomic data has enabled researchers to go a step further and integrate big data from different kinds of -omics analyses to address fundamental questions. Over 120 participants gathered at the Plant Genomes & Biotechnology: From Genes to Networks meeting to discuss novel findings in diverse areas of plant biology using one or multiple -omics techniques. Overall, the speakers underlined the advantages of multi-omics tools exploring the diversity in physiological processes across plants, from model organisms and crops to carnivorous plants. While the multi-omics approach has many advantages, it could also present itself as overwhelming in both data quantity and complexity. Many talks presented at this meeting illustrated ways to approach big data to answer both general and detailed questions in plant biology. Indeed, it was evident that the questions that can be tackled using -omics approaches are vastly different from characterizing a single gene in a specific developmental or immune pathway. Rather, -omics approaches facilitate viewing and understanding particular biological processes as part of a larger enterprise: the whole plant and its connection with the environment. The meeting offered fresh insights from applying -omics to discovery, in the fields of abiotic and biotic stress response, epigenetics and genetics, hormone signaling, growth and development, biodiversity and adaptation to environment, and synthetic and network biology. Some of the most thought-provoking uses of multi-omics techniques presented at the conference are discussed below and include the characterization of intergenic regions, defining genes and pathways involved in specific processes, and measuring dynamic responses in tissues, whole plants or plant populations.

## Beyond gene annotation: understanding transcriptional control

The number and quality of plant genomes produced in recent years have been staggering. Jason Williams (Cold Spring Harbor Laboratories, USA) reported that between 50 and 80 plant genomes have been sequenced in 2013 alone. This flux of genomic and transcriptomic data is challenging to store and share between users, which is why Williams and associates have developed iPlant as a platform for data storage and analysis. Mining genomic data has already provided novel insights for understanding how and when plants turn on genes. The keynote speaker, Joe Ecker (Salk Institute, USA), has made considerable contributions in understanding hormonal-mediated transcriptional regulation. By combining data from transcription factor binding sites with methylation data and transcriptomes, Ecker showed that ethylene induces waves of gene induction, targeting all of the other plant hormone pathways. A number of other studies also integrated similar tools to expand our current understanding of gene expression. For example, the complexity of gene induction was outlined by a study of 26 transcription factors presented by Ken Heyndrickx (Ghent University, Belgium). Heyndrickx demonstrated that transcription factors bind multiple motifs and that binding does not correlate with expression, drawing in to question how gene expression is regulated. Further insights into the importance and complexity of intergenic regions were provided by Luis Herrera-Estrella (National Polytechnic Institute, Mexico) in his investigation of a carnivorous underwater plant called *Utricularia gibba*. The genome of *U. gibba* is small at only 82 megabases, although it contains approximately similar numbers of genes as other plants. The difference in size is mainly due to reduced intergenic DNA, which begs the question: what are the requirements for intergenic DNA length to drive gene expression in plants? Herrera-Estrella demonstrated that while *U. gibba* intergenic regions are small compared with other plants, they are sufficient to drive gene expression.

## Plants in relation to their environment

Several participants reported the use of multi-omics tools in understanding developmental processes or stress responses. A range of multi-omics approaches included the integration of transcriptomics, metabolomics, phenomics or interactomics. In a particularly thorough study, Hilde Nelissen (Ghent University, Belgium) described a study of the maize leaf transition zone to characterize the role of gibberellins in plant growth. Using information on gene transcription, protein interactions, hormone accumulation and microscopy in growing sections of the maize leaf, Nelissen identified growth-related genes that when overexpressed were shown to increase plant growth. The power of phenomics in producing rapid and sensitive analysis of phenotypes over time was also impressive. Techniques describing large-scale analysis of photosynthetic parameters, inflorescence architecture, root growth and field growth performance were presented. This approach, when combined with genomics and reverse genetics, has the potential to quickly identify genes responsible for subtle phenotypes, which might otherwise be overlooked due to constraints of time and money. To understand where new genomic variation originates, Detlef Weigel (Max-Planck-Institute for Developmental Biology, Germany) measured the mutation accumulation rate in 30 generations of *Arabidopsis*. Weigel determined that while the mutation rate per generation is reduced, the rate becomes significant when considering an entire plant population.

## Studying the dynamics of life

Plants are dynamic living systems wherein external and internal signals are integrated to produce changes over time. While valuable information can be gleaned from the functional role of a given gene at one time point, combining reverse genetics with multi-omics can better capture how biological processes work temporally. For example, Ross Sozzani (North Carolina State University, USA) developed an elegant system to image root stem cell dynamics *in vivo*. By combining visualization of auxin and *SHORTROOT* transcript gradients, Sozzani described a system whereby the movement of the visualized transcripts could be related to cell division rates. Detecting phenotypes in mutant lines can also benefit from temporal measurements, especially in the context of environmental response. David Kramer (Michigan State University, USA) was able to detect a photosynthetic phenotype in 20% of his screened *Arabidopsis* T-DNA insertion mutants during light fluctuations, while only 2% of these lines demonstrated a phenotype without light fluctuations. Methods to study protein dynamics are essential for mapping plant interactomes. A high-throughput protein complex purification platform developed by Geert de Jaeger (Ghent University, Belgium) has generated a dynamic map of protein-protein interactions assembled during the cell cycle, and generated novel insights into this critical process. A method for the detection of protein interactions in plant cells, presented by Dharmendra Singh (The Boyce Thompson Institute, USA), has shown a potential for discovering associations between plant and pathogen proteins on a large scale.

## Predicting the future

One of the main objectives in plant biology is to reliably predict plant responses given genomic information and environmental circumstances. The copious amount of data derived from multi-omics studies has the potential to contribute to this goal. Indeed, plant modeling tools are already available, and multi-omics techniques are already being used to speed up plant breeding. However, there is still much to be discovered, and the talks presented at this meeting underscore how multi-omics approaches are changing plant research. The nature of -omics analyses shifts research time from taking measurements to analyzing big data and finding relevant trends. Big data also expands the questions we can ask to include niches or processes that have thus far been too complex to study in depth. A great example is the characterization of the ecology of the root bacteriome across soil conditions presented by Sarah Lebeis (University of North Carolina, USA). While the study of this system still requires extensive wet lab analysis, Lebeis used big data as a platform to begin her investigation into root bacterial communities and their effect on plant growth. Thus, carefully generated big data can guide biological discovery and complement targeted approaches in dissecting plant processes. Multi-omics approaches unify knowledge on the function and regulation of individual genes/pathways with contextual information - in short, it is a step forward toward capturing the essence of a plant.

## Competing interests

The authors declare that they have no competing interests.

